# Disrupted Bone Metabolism in Long-Term Bedridden Patients

**DOI:** 10.1371/journal.pone.0156991

**Published:** 2016-06-08

**Authors:** Keiko Eimori, Naoto Endo, Seiji Uchiyama, Yoshinori Takahashi, Hiroyuki Kawashima, Kei Watanabe

**Affiliations:** 1 Divisions of Pediatric Orthopedic Surgery, Nishi-Niigata Chuo National Hospital, Niigata, Japan; 2 Department of Regenerative and Transplant Medicine, Division of Orthopedic Surgery, Niigata University Graduate School of Medical and Dental Sciences, Niigata, Japan; 3 Divisions of Orthopedic Surgery, Nishi-Niigata Chuo National Hospital, Niigata, Japan; Leeds Beckett University, UNITED KINGDOM

## Abstract

**Background:**

Bedridden patients are at risk of osteoporosis and fractures, although the long-term bone metabolic processes in these patients are poorly understood. Therefore, we aimed to determine how long-term bed confinement affects bone metabolism.

**Methods:**

This study included 36 patients who had been bedridden from birth due to severe immobility. Bone mineral density and bone metabolism markers were compared to the bedridden period in all study patients. Changes in the bone metabolism markers during a follow-up of 12 years were studied in 17 patients aged <30 years at baseline.

**Results:**

The bone mineral density was reduced (0.58±0.19 g/cm^3^), and the osteocalcin (13.9±12.4 ng/mL) and urine N-terminal telopeptide (NTX) levels (146.9±134.0 mM BCE/mM creatinine) were greater than the cutoff value for predicting fracture. Among the bone metabolism markers studied, osteocalcin and NTX were negatively associated with the bedridden period. During the follow-up, osteocalcin and parathyroid hormone were decreased, and the 25(OH) vitamin D was increased. NTX at baseline was negatively associated with bone mineral density after 12 years.

**Conclusions:**

Unique bone metabolic abnormalities were found in patients who had been bedridden for long periods, and these metabolic abnormalities were altered by further bed confinement. Appropriate treatment based on the unique bone metabolic changes may be important in long-term bedridden patients.

## Introduction

Because mechanical stress is important for maintaining bone mass, immobilization for prolonged periods results in bone atrophy and osteoporosis [1–3]. Fractures are often observed in children and adults with severe immobility who are bedridden for long periods due to neuromuscular diseases, such as cerebral palsy, epilepsy, and encephalitis [4–6]. In such immobilized patients, low bone mineral density (BMD) and abnormal bone metabolism have been reported [7–12]. However, the available evidence showing long-term changes in the BMD and bone metabolism has been limited. The life expectancy of patients with severe neuromuscular diseases has continually increased due to improvements in medical treatment and care [13,14]. As a result, painful bone fractures remain one of the most serious complications of being bedridden, and the importance of maintaining bone health is increasing [14–16]. Therefore, we investigated how bed confinement for a prolonged period affects bone metabolism in patients with severe motor and intellectual disabilities, none of whom have stood or walked in their life.

## Materials and Methods

### Patients

This study included patients with severe immobility who had never had the ability to stand or walk in the Severe Motor and Intellectual Disability Center at the Nishi-Niigata Chuo National Hospital, Japan. Informed consent to participate in this study was obtained from the guardians of the participants. Because all of the participants have severe intellectual disability, and they could not judge if our study is acceptable. The study protocol and this consent procedure were approved by the Ethical Committee of the Nishi-Niigata Chuo National Hospital (approval number, 1022). Patients who were <18 years and those who were ≥45 years in 1999 were excluded. Bone metabolism markers and the BMD of the lumbar spine were evaluated in both 1999 and 2011.

### Measurements

The BMD of lumbar spine 2–4 in the anteroposterior projection was assessed using dual energy X-ray absorptiometry (DXA) (Hologic, Bedford, MA, USA). Radiologists checked for spinal deformities via the radiography of the lumbar spine and then used the frontal view of lumbar spine 2–4 to provide an accurate evaluation of BMD. For the evaluation of bone metabolism, the levels of serum osteocalcin, urine N-terminal telopeptide (NTX), serum 25-hydroxy vitamin D (25(OH) vitamin D), serum intact parathyroid hormone (PTH), serum calcium, serum phosphorus, and serum alkaline phosphatase were measured. The NTX levels were measured using an enzyme immunoassay (EIA) (BML, Tokyo, Japan), the osteocalcin levels were measured using an immune radiometric assay (IRMA) (BML), the 25(OH) vitamin D levels were measured using a radio immunoassay (RIA) (SRL, Tokyo, Japan), and the intact PTH levels were measured using an electrochemiluminescence immunoassay (ECLIA) (SRL). Serum calcium, phosphorus, and alkaline phosphatase levels were measured by automated standard laboratory methods. Blood and urine samples were collected in a fasting state before breakfast. For the evaluation of BMD levels, the cutoff value (0.708 g/cm^3^) proposed for osteoporosis based on the guidelines for osteoporosis by the Japan Osteoporosis Society, the Japanese Society for Bone and Mineral Research, and the Japanese Osteoporosis Foundation was used [17]. For the evaluation of osteocalcin and NTX levels, the cutoff values (4.5 ng/mL for osteocalcin and 54.3 nM BCE/mM Cr for NTX) proposed for predicting fracture based on the guidelines were used [17].

### Statistical analysis

The effects of long bedridden periods on bone metabolism were studied using both a cross-sectional analysis based on data from 1999, and a longitudinal analysis based on data from 1999 and 2011. In the cross-sectional analysis, the BMD and bone metabolism markers were compared with the bedridden period using linear regression analysis in all study patients. In the longitudinal analysis, changes in the bone metabolism markers from 1999 to 2011 were compared using a paired t-test in patients aged <30 years in 1999. Furthermore, the effects of bone metabolism markers in 1999 on BMD in 2011 were studied using linear regression analysis. A two-sided P<0.05 was considered statistically significant. Statistical analyses were performed using SPSS for Windows version 21 (IBM Inc., Armonk, NY, USA).

## Results

This study included 36 patients (17 females [47%]; mean age, 32±7 years) (Table 1). Cerebral palsy was the most common reason for patients to be bedridden (n = 23 [64%]) (Table 2). The serum albumin levels (4.3±0.4 g/dL) were within the normal range, and no patient exhibited apparent undernutrition. More than half of the patients received antiepileptic drugs, and no patients had undergone previous surgical treatment. Four patients had a history of fracture; three patients had a history of lumbar spine fracture and one patient had a history of femoral fracture. There was no significant difference in the baseline clinical characteristics among the causes of bedrest (Table 2).

**Table 1:** Clinical characteristics in all study participants (n = 36).

Female sex, n (%)	17 (47)
Age, years	32±7
Height, cm	139.8±13.1
Weight, kg	28.5±7.0
Body mass index	14.5±2.8
Serum albumin, g/dL	4.3±0.4
Antiepileptic drugs, n (%)	25 (69)

**Table 2:** Comparisons of clinical characteristics by underlying diseases.

Underlying diseases	n (%)	Female sex, n (%)	Age, years	Body mass index	Serum albumin, g/dL	Antiepileptic drugs, n (%)	Bone mineral densit, g/cm^2^
Cerebral palsy	23 (64)	11 (48)	35±6	14.6±3.0	4.3±3.0	14 (61)	0.65±0.22
Encephalitis	4 (11)	2 (50)	31±5	15.4±1.4	4.2±0.3	3 (50)	0.7±0.26
Head injury	3 (8)	1 (33)	24±4	14.2±3.3	4.2±0.8	3 (100)	0.61±0.17
Others*	6 (17)	3 (50)	27±5	13.9±2.5	4.4±0.5	5 (83)	0.61±0.13

There was no difference in the clinical characteristics among these groups.

* Others include hydrocephaly (n = 2), epilepsy (n = 2), rett syndrome (n = 1) and hyperphenylalaninemia (n = 1)

**Fig 1:**
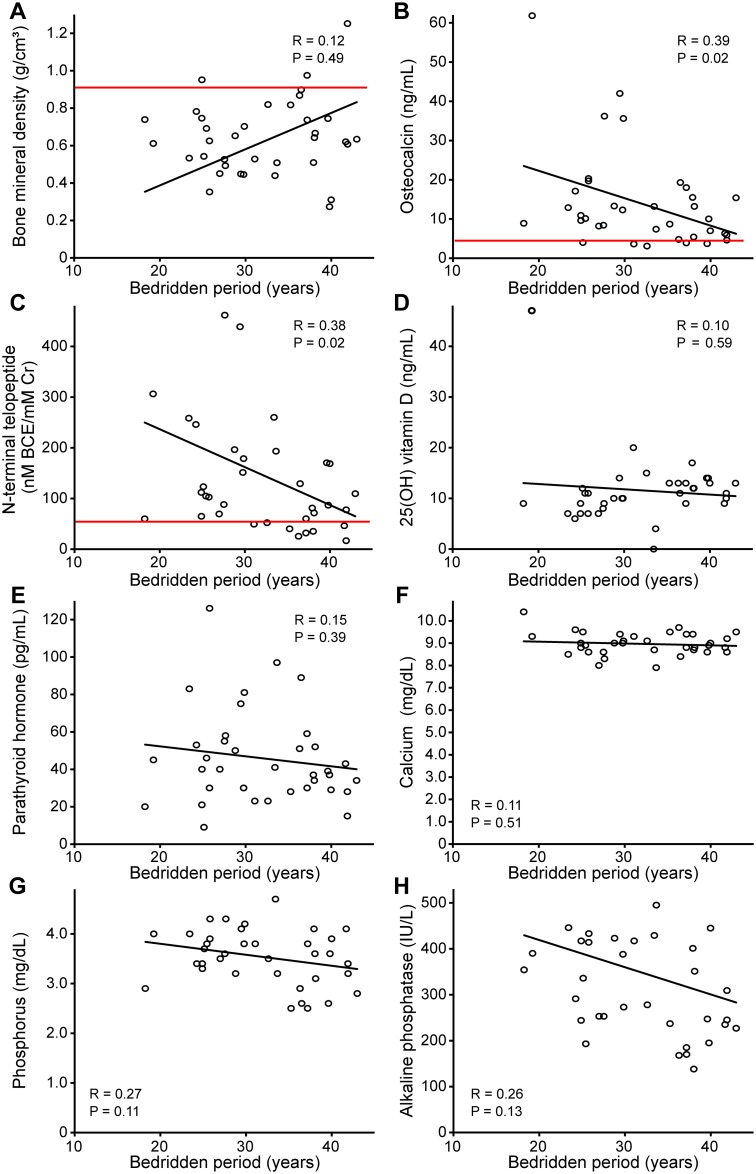
Bone mineral density and bone metabolism marker levels according to the period of bed confinement in all study patients. Because all patients had been bedridden from birth, the period of bed confinement was equal to their age. A) Correlation between the bone mineral density of lumbar spine vertebrae 2–4 in the anteroposterior projection and the period of bed confinement. Correlation between the bone metabolism marker levels, including the serum osteocalcin levels (B), urine N-terminal telopeptide levels (C), serum 25 (OH) vitamin D levels (D), serum intact parathyroid hormone levels (E), serum calcium levels (F), serum phosphorus levels (G), and serum alkaline phosphatase levels (H), and period of bed confinement. Red lines indicate the cutoff value for osteoporosis (bone mineral density) and those for predicting bone fracture (osteocalcin and N-terminal telopeptide), as proposed by the guidelines for the prevention and treatment of osteoporosis [17].

The results of the cross-sectional analysis based on data from 1999 in all 36 study patients are shown in Table 3 and Fig 1. In 24 patients (67%), the BMD levels were reduced below the cutoff value for osteoporosis (0.708 g/cm^3^) based on the Japanese guidelines for osteoporosis (Fig 1) [17]. There was no difference in the BMD levels among the causes of bedrest (Table 2). The serum osteocalcin levels were greater than the cutoff value for predicting fracture (4.5 ng/mL) based on the guidelines in 30 of 36 patients (83%), and the urine NTX levels were greater than the cutoff value for predicting fracture (54.3 nM BCE/mM Cr) in 28 patients (78%) [17]. Among the bone metabolism markers, the serum osteocalcin and urine NTX levels were negatively correlated with a bedridden period (Fig 1B and 1C). However, other bone metabolism markers were not correlated with the bedridden period (Fig 1).

**Table 3:** Bone mineral density and bone metabolism marker levels in all study patients.

Bone mineral density, g/cm^2^*	0.58±0.19
Calcium, mg/dL^†^	9.0±0.5
Phosphorus, mg/dL	3.5±0.6
Alkaline phosphatase (IU/L)	348±157
25(OH) vitamin D, ng/ml	11.9±6.9
Parathyroid hormone, pg/mL	45.9±25.0
Osteocalcin ng/mL	13.9±12.4
Urine N-terminal telopeptide, nM BCE/nM creatinine	146.9±134.0

* Bone mineral density of lumbar spine 2–4 in anteroposterior projection and normal value based on diagnostic criteria for osteoporosis by Japanese Society of Bone and Mineral Research.

^†^ Serum calcium adjusted by albumin

**Fig 2:**
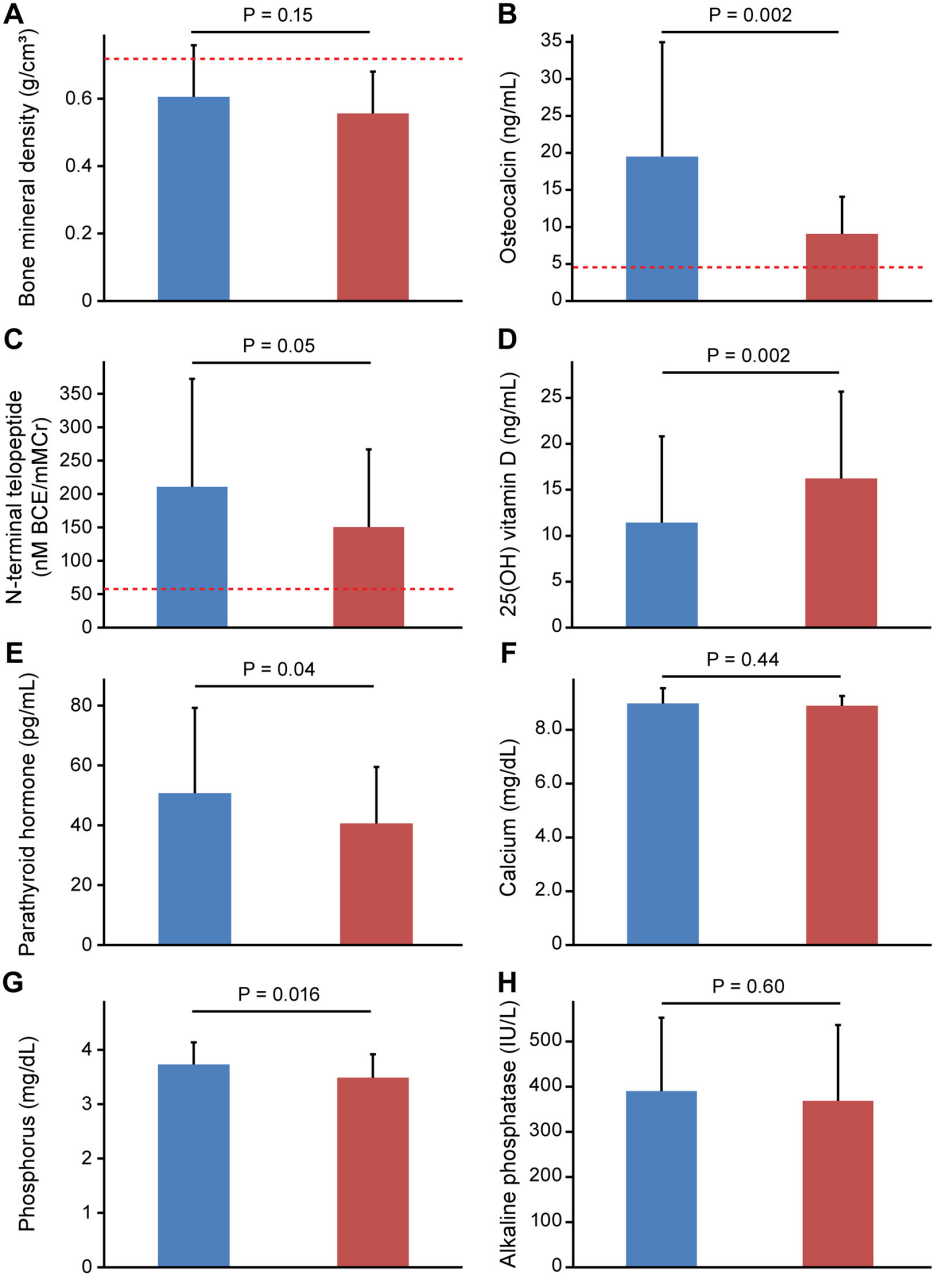
Changes in the bone mineral density and bone metabolism marker levels during a 12-year follow-up period in patients whose baseline age was <30 years. Red lines indicate the cutoff value for osteoporosis (bone mineral density) and those for predicting bone fracture (osteocalcin and N-terminal telopeptide), as proposed by the guidelines for the prevention and treatment of osteoporosis [17].

**Fig 3:**
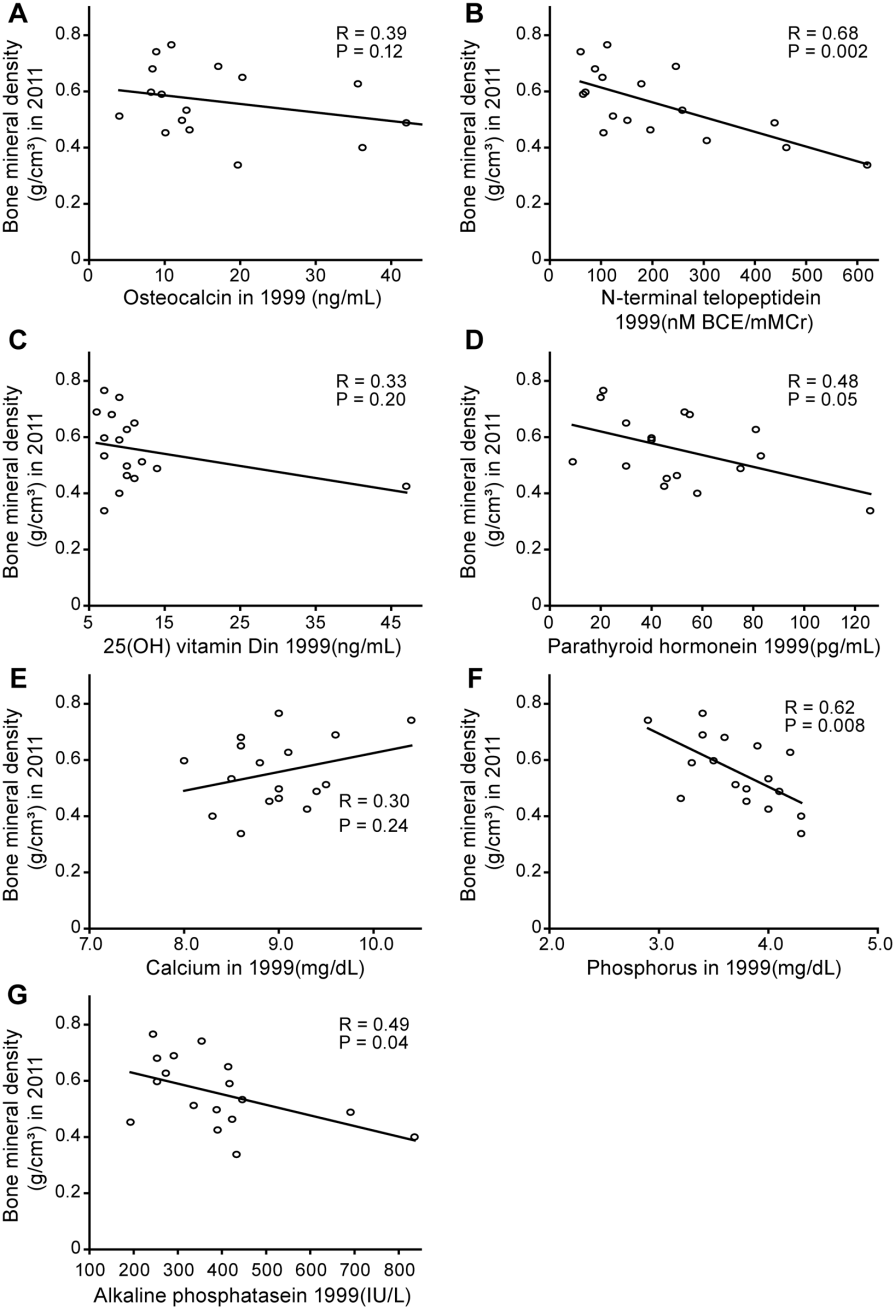
Association between the bone metabolism marker levels at baseline with the bone mineral density during a follow-up of 12 years in patients whose baseline age was <30 years.

The clinical characteristics in 17 patients aged <30 years in 1999 are shown in Table 4. The patients included 8 females (47%), with a mean age of 25.7±3.3 years. The results of the longitudinal analysis based on data from 1999 and 2011 in 17 patients aged <30 years in 1999 are shown in Table 5 and Figs 2 and 3. During a follow-up of 12 years from 1999 to 2011, two patients with osteoporosis developed fractures; one patient whose BMD level was 0.53 g/cm^3^ had rib fracture and another one whose BMD level was 0.63 g/cm^3^ had humeral neck fracture. Although the change in the BMD levels during the follow-up period was not statistically significant (Table 5, Fig 2A), the number of patients with the reduced BMD level, which was less than the cutoff value for osteoporosis, was increased from 13 patients (76%) in 1999 to 15 patients (88%) in 2011.17 During the follow-up period, the osteocalcin levels had decreased (Fig 2A), but the number of patients with osteocalcin levels greater than the cutoff value for osteoporosis was still high (16 patients [94%] in 1999; 15 patients [88%] in 2011). The urine NTX levels tended to decrease (Fig 2C), and the number of patients with BMD values less than the cutoff had decreased (17 patients [100%] in 1999; 12 patients [71%] in 2011). Among other bone metabolism markers, the 25(OH) vitamin D levels had increased (Fig 2D), and the parathyroid hormone (Fig 2E) and phosphorus levels (Fig 2G) had decreased during the follow-up.

**Table 4:** Baseline characteristics in 17 patients <30 years followed for 12 years.

Female sex, n (%)	8 (47)
Age, years	25±3
Height, cm	136.8±13.3
Weight, kg	26.8±6.8
Body mass index	14.2±2.4
Serum albumin, g/dL	4.3±0.5
Underlying diseases, n (%)	
Cerebral palsy	8 (47)
Encephalitis	2 (12)
Head Injury	3 (18)
Epilepsy	2 (12)
Antiepileptic drugs, n (%)	13 (77)

**Table 5:** Changes in bone metabolism markers during long bedridden periods.

	Baseline	After 12 years	P value
Bone mineral density, g/cm^2^*	0.61±0.15	0.56±0.12	0.15
Calcium, mg/dL^†^	9±0.6	8.9±0.4	0.44
Phosphorus, mg/dL	3.7±0.4	3.5±0.4	0.016
Alkaline phosphatase (IU/L)	390±163	368±168	0.60
25(OH) vitamin D, ng/mL	11.4±9.4	16.2±9.5	0.002
Parathyroid hormone, pg/mL	50.7±28.5	40.6±18.9	0.038
Osteocalcin, ng/mL	19.5±15.5	9.0±5.0	0.002
Urine N-terminal telopeptide, nM BCE/mM creatinine	210.7±161.7	150.4±116.5	0.052

* Bone mineral density of lumbar spine 2–4 in anteroposterior projection and normal value based on diagnostic criteria for osteoporosis by Japanese Society of Bone and Mineral Research.

^†^ Serum calcium adjusted by albumin

Fig 3 shows the effects of the bone metabolism marker levels in 1999 on the BMD levels in 2011. The urine NTX (Fig 3B), phosphorus (Fig 3D), and alkaline phosphatase levels (Fig 3G) in 1999 were negatively associated with the BMD levels in 2011. The parathyroid hormone levels in 1999 tended to be associated with the BMD levels in 2011 (Fig 2D). However, the osteocalcin levels in 1999 were not associated with the BMD levels in 2011 (Fig 3A). Although the correlations of low nutritional status and anticonvulsants with low BMD have been reported in the previous cross-sectional studies [18,19], the serum albumin levels and use of anticonvulsants in 1999 were not associated with the BMD levels in 2011 in this longitudinal study (R = 0.048 and P = 0.85 for albumin levels, R = 0.06 and P = 0.81 for anticonvulsant use).

## Discussion

In this study, patients who had been bedridden from birth exhibited low BMD and abnormal bone metabolism. In long-term bedridden patients, the osteocalcin and NTX levels were high at a relatively young age, but the levels of both bone metabolism markers had decreased thereafter. The high levels of NTX, phosphorus, and alkaline phosphatase at baseline were associated with decreased BMD during the 12-year follow-up.

Mechanical stress is important for maintaining bone mass, and mechanical unloading, such as long-term bed confinement, leads to osteoporosis. In addition to elderly adults whose daily activity is limited, children and young adults with severe immobility are at a high risk of developing osteoporosis [7–12,19,20]. Reduced BMD has been reported in patients with neuromuscular diseases such as cerebral palsy, spastic quadriplegia, and epilepsy [7–12,19,20]. Furthermore, the severity of physical disability has been associated with reduced BMD [12,18]. This study included patients who had been bedridden from birth and showed that the BMD was highly reduced in such patients. Although the causes of immobility may affect the severity of osteoporosis, there was no difference in the BMD levels among the underlying neuromuscular diseases.

Increased bone resorption during immobilization periods can lead to bone fragility [21,22]. In this study, NTX, a marker of bone resorption, was greater than the cutoff value proposed for predicting fracture in the majority of patients [17]. Osteocalcin, a marker of bone formation, was also greater than the cutoff value proposed for predicting fracture in the majority of patients. In line with these findings of this study, there is a close correlation between NTX and osteocalcin in patients with cerebral palsy in a previous study [19]. Both NTX and osteocalcin had decreased but were still found at high levels during a follow-up of 12 years in patients whose baseline age was between 18 and 30 years. These findings suggest that bone turnover is accelerated until a relatively young age in immobilized patients, after which the acceleration decreases, although the majority of patients still have accelerated bone turnover.

There are several risk factors for osteoporosis in immobilized patients. In previous cross-sectional studies, severe motor impairment, less physical activity, low nutritional status, and low calcium intake are all correlated with low BMD [11,18,19]. In contrast, serum calcium, alkaline phosphatase, osteocalcin, and 25(OH) vitamin D are not correlated with BMD [12,18]. The role of anticonvulsants in low BMD is controversial, and in this study, the use of anticonvulsants was not associated with BMD [11,18]. In this longitudinal study, high NTX levels at baseline were associated with reduced BMD during a follow-up period of 12 years in patients aged between 18 and 30 years. However, NTX was not correlated with BMD in patients with cerebral palsy aged <19 years in a previous cross-sectional study [19]. Taken together, increased bone resorption seems to have an important role in osteopenia in patients who had been bedridden for a very long period from birth due to severe immobility. In this study, high NTX levels at baseline were associated with reduced BMD during the follow-up period. Evidence that high levels of phosphorus and alkaline phosphate, both of which indicate increased bone turnover, were associated with reduced BMD during the follow-up period further supports our hypothesis, although alkaline phosphate was not found to be correlated with BMD in a previous cross-sectional study [18]. NTX, phosphorus, and alkaline phosphate may be useful in predicting the development of osteoporosis in the future.

This study had several limitations. The patients were affected by severe motor and intellectual disabilities and had various underlying diseases, and our results may not apply to all bedridden patients. The number of patients was small, and additional analyses could not be sufficiently performed in subgroups based on the underlying diseases. Data for bone metabolism markers were absent in patients during the growth period. Nevertheless, our findings are remarkable because the study participants had been bedridden for very long periods, which has not been studied previously. Additional studies involving larger numbers of patients will be necessary to uncover the mechanisms of osteoporosis and to improve treatments for osteoporosis in long-term bedridden patients.

In conclusion, bone formation and resorption were found to be accelerated in patients with <30 years of bed confinement, followed by a decrease in accreted bone metabolites. Increased bone resorption was associated with a low BMD during the follow-up period. Appropriate treatment based on changes in bone metabolism may be important for the prevention of bone fracture in long-term bedridden patients.
